# Whence CRIPTO: The Reemergence of an Oncofetal Factor in ‘Wounds’ That Fail to Heal

**DOI:** 10.3390/ijms221810164

**Published:** 2021-09-21

**Authors:** David W. Freeman, Elisa Rodrigues Sousa, Sofia Karkampouna, Eugenio Zoni, Peter C. Gray, David S. Salomon, Marianna Kruithof-de Julio, Benjamin T. Spike

**Affiliations:** 1 Department of Oncological Sciences, School of Medicine, University of Utah, Salt Lake City, UT 84113, USA; David.Freeman@hci.utah.edu; 2 Urology Research Laboratory, Department for BioMedical Research DBMR, University of Bern, 3012 Bern, Switzerland; elisa.rodriguessousa@dbmr.unibe.ch (E.R.S.); sofia.karkampouna@dbmr.unibe.ch (S.K.); Eugenio.zoni@dbmr.unibe.ch (E.Z.); 3 Peptide Biology Laboratory, The Salk Institute for Biological Studies, La Jolla, CA 92037, USA; gray@salk.edu; 4 Mouse Cancer Genetics Program, Center for Cancer Research, National Cancer Institute, Frederick, MD 20893, USA; salomond@mail.nih.gov; 5 Translational Organoid Models, Department for BioMedical Research, University of Bern, 3012 Bern, Switzerland; 6 Bern Center for Precision Medicine, Inselspital, University Hospital of Bern, 3010 Bern, Switzerland; 7 Department of Urology, Inselspital, University Hospital of Bern, 3010 Bern, Switzerland

**Keywords:** CRIPTO, stem cells, EMT, cancer, metastasis, fibrosis, therapeutic target

## Abstract

There exists a set of factors termed oncofetal proteins that play key roles in ontogeny before they decline or disappear as the organism’s tissues achieve homeostasis, only to then re-emerge in cancer. Although the unique therapeutic potential presented by such factors has been recognized for more than a century, their clinical utility has yet to be fully realized1. This review highlights the small signaling protein CRIPTO encoded by the tumor derived growth factor 1 (*TDGF1*/*Tdgf1*) gene, an oft cited oncofetal protein whose presence in the cancer literature as a tumor promoter, diagnostic marker and viable therapeutic target continues to grow. We touch lightly on features well established and well-reviewed since its discovery more than 30 years ago, including CRIPTO’s early developmental roles and modulation of SMAD2/3 activation by a selected set of transforming growth factor β (TGF-β) family ligands. We predominantly focus instead on more recent and less well understood additions to the CRIPTO signaling repertoire, on its potential upstream regulators and on new conceptual ground for understanding its mode of action in the multicellular and often stressful contexts of neoplastic transformation and progression. We ask whence it re-emerges in cancer and where it ‘hides’ between the time of its fetal activity and its oncogenic reemergence. In this regard, we examine CRIPTO’s restriction to rare cells in the adult, its potential for paracrine crosstalk, and its emerging role in inflammation and tissue regeneration—roles it may reprise in tumorigenesis, acting on subsets of tumor cells to foster cancer initiation and progression. We also consider critical gaps in knowledge and resources that stand between the recent, exciting momentum in the CRIPTO field and highly actionable CRIPTO manipulation for cancer therapy and beyond.

## 1. Introduction

### Epigraphs

*“Elements which have retained their […] embryonal characteristics in the adult organism or have regained them through some chemico-physiologic deviation, represent […] the generative elements of every tumor variety and specifically those of a malignant nature. Such elements may remain enclosed within matured tissues for many years, giving no indication of their presence, until an irritation—a simple stimulus suffices—rekindles their vital cellular activities…”*

—Mueller, 1874 [1].

*“Could we but learn from whence his sorrows grow, we would as willingly give cure as know.”*

—William Shakespeare.

There exists a set of factors termed oncofetal proteins that play key roles in ontogeny before they decline or disappear as the organism’s tissues achieve homeostasis, only to then re-emerge in cancer. Although the unique therapeutic potential presented by such factors has been recognized for more than a century, their clinical utility has yet to be fully realized [2]. This review highlights the small signaling protein CRIPTO (CRIPTO-1; CR-1; TDGF1) encoded by the tumor derived growth factor 1 *(TDGF1/Tdgf1)* gene, an oft cited oncofetal protein whose presence in the cancer literature as a tumor promoter, diagnostic marker and viable therapeutic target continues to grow. We touch lightly on features well established (and well-reviewed [3,4,5,6,7]) since its discovery more than 30 years ago, including CRIPTO’s early developmental roles and modulation of SMAD2/3 activation by a selected set of transforming growth factor β (TGF-β) family ligands. We predominantly focus instead on more recent and less well understood additions to the CRIPTO signaling repertoire, on its potential upstream regulators and on new conceptual ground for understanding its mode of action in the multicellular and often stressful contexts of neoplastic transformation and progression. We ask whence it re-emerges in cancer and where it ‘hides’ between the time of its fetal activity and its oncogenic reemergence. In this regard, we examine CRIPTO’s restriction to rare cells in the adult, its potential for paracrine crosstalk, and its emerging role in inflammation and tissue regeneration—roles it may reprise in tumorigenesis, acting on subsets of tumor cells to foster cancer initiation and progression. We also consider critical gaps in knowledge and resources that stand between the recent, exciting momentum in the CRIPTO field and highly actionable CRIPTO manipulation for cancer therapy and beyond.

## 2. Rise and Fall of CRIPTO

### 2.1. CRIPTO Regulates Undifferentiated Cellular Phenotypes during Development

**Figure 1:**
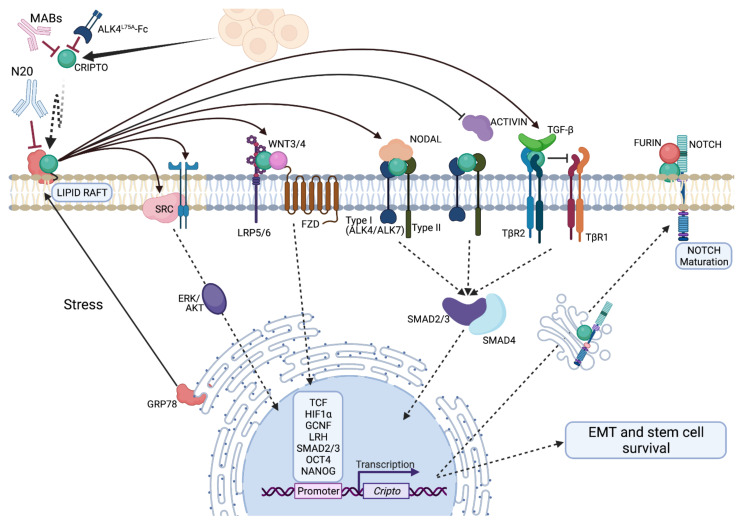
Molecular signaling mechanisms of CRIPTO. Created in BioRender 15 August 2021.

CRIPTO is the founding member of the EGF-CFC protein family whose members share the same general structure including an epidermal growth factor (EGF)-like domain; a cysteine rich, family specific Cr1-Frl1-cryptic (CFC) domain; and a hydrophobic C-terminus which can serve as a GPI-anchor sequence [8,9,10,11]. Members of the EGF-CFC protein family are highly conserved across species and include human and mouse CRIPTO (Cr-1, Cripto-1, TDGF1); cryptic (CFC/Cfc), *Xenopus* FRL1 and zebrafish one-eyed pinhead (oep) as well as members in several invertebrates including sea urchin and lancelet, illustrating a high degree of evolutionary conservation [9,12,13,14]. These orthologs play a major role in early body axis patterning. Constitutive CRIPTO knockout is embryologically lethal as *Tdgf1*-null mice fail to undergo gastrulation due to an inability to develop proper anterior-posterior polarity [15]. Constitutive germline knockout of CRIPTO in mouse epiblasts during gastrulation disrupts primitive streak formation and subsequently impairs migration of mesodermal and endodermal precursors [16]. Similar early patterning defects are seen in zebrafish *Oep* knockouts. These zebrafish defects are attenuated with injection of mouse *Tdgf1* mRNA underscoring the high level of conserved functionality between these orthologs [9]. The effects of these orthologs during early gastrulation are driven through their defined roles as essential co-receptors that facilitate binding of a subset of TGF-β superfamily members including NODAL, GDF1 and GDF3 to their signaling receptors and activation of downstream SMAD2/3 signaling [17,18,19]. Additionally, CRIPTO has also been reported to attenuate signaling by other TGF-β superfamily members, namely ACTIVIN A, ACTIVIN B and TGF-β1 and downstream SMAD2/3 signaling through distinct mechanisms (Figure 1) [20,21,22].

CRIPTO expression is tightly linked to primitive stem cell states. Meta-studies of transcriptional profiles from mouse embryonic stem cells (ESC) and induced pluripotent stem cells (iPSC) identified *Tdgf1* among the most consistently expressed genes across multiple studies [23,24]. And a study of heterogeneity within ESC cultures identified CRIPTO expression as a mark of the most primitive cells with the most plasticity and proliferative potential [25]. Indeed, Yamanaka’s landmark paper on cellular reprogramming used *Tdgf1* as a hallmark of complete reprogramming [26]. In the intact epiblast in vivo, CRIPTO protein and RNA are expressed in a salt-and-pepper pattern in the subset of inner cell mass cells exhibiting the highest levels of nuclear NANOG [27]. CRIPTO is critical for maintenance of pluripotency, regulating ACTIVIN/NODAL signaling in mouse Epiblast Stem cells (EpiSC), the cellular counterparts of cultured human ESCs [27]. Additionally, ESC renewal in mice is mediated by CRIPTO modulation of Wnt/β-catenin signaling [27]. Comparative analysis of cultured ESCs from mice and humans shows highly correlated expression of *Tdgf1* with core ESC regulatory circuit genes including the signature genes *Sox2*, *Oct-4*, *Lefty*, *Utf-1* and *Dnmtl*, and CRIPTO may be functionally integral to the stem cell state through reinforcement of the core stem cell circuit [28]. Indeed, binding sites for both NANOG and OCT-4 were identified by ChIP-PET within the *Tdgf1* promoter and repression of *Nanog* and *Oct-4* in ESCs significantly reduces *Tdgf1* expression [29]. 

Outside the inner cell mass, functional requirements for CRIPTO are maintained in the earliest distinctive differentiation steps of development as CRIPTO has been detected and determined to be functionally required in trophoblast cells that generate fetal derived placental tissue [30]. In this context, as in early axis patterning processes, CRIPTO appears to control cellular differentiation and migration [31]. Interestingly, CRIPTO is also required from the maternal side of placenta formation where it regulates vascularization and may regulate the reacquisition of some precursor adult cell that is required for embryo implantation in the uterus [30,32].

### 2.2. CRIPTO Hides

The apparent loss of CRIPTO in adult tissues that has contributed to its recognition as an oncofetal protein likely reflects the rarity of the cells expressing it (rather than its absence per se). Normally, CRIPTO appears restricted to stem cell niches and contexts that call upon its re-expression in a small subpopulation of adult somatic cells including certain regenerative processes (summarized in Table 1). Thus, CRIPTO has been implicated in the stem cell compartment of hierarchically organized tissues including the gastrointestinal epithelium, hematopoietic system and breast, in normal cyclic regenerative processes in such tissues (especially adult mammary and endometrial tissues), and in injury/regeneration settings in muscle, cartilage, pancreatic, cardiac, and hepatic tissues [6,33,34,35,36,37,38]. 

CRIPTO message was detected in adult colonic stem cells which represent a small minority of cells in the crypts that lie at the base of colonic villi alongside well-studied intestinal stem cell markers like *Lgr5*, though CRIPTO’s function there has not been assessed [68,69]. CRIPTO was also detected in specialized regions of the bone marrow where hematopoietic stem cells (HSCs) are found in an osteoid niche, and in this context CRIPTO was shown to play a critical role in maintenance of the HSC state [39]. Interestingly, the osteoid niche involves the coordinated activity of hypoxia inducible factor 1-alpha (HIF1α and stromal cells whose specific identities remain to be fully delineated [33,39]. The involvement of HIF1α in CRIPTO’s regulation of HSCs reflects a recurrent theme wherein integration of micro-environmental stress (often hypoxia but perhaps other stress signals) and CRIPTO-dependent SMAD and growth factor (GF)-like signaling lead to maintenance or facultative expansion of stem cell function.

In the mammary gland, early tissue level immunodetection studies showed CRIPTO is regulated during tissue remodeling, correlating with hormonally regulated tissue remodeling and being most pronounced during mammary alveolar development [43,44,70]. Consistent with a functional role in alveolar remodeling, conditional knockout of CRIPTO in the mammary epithelium, under control of whey acidic promoter (WAP) showed significant reduction in alveolar development during pregnancy and a subsequent delay in involution possibly indicating a role for CRIPTO specifically in luminal progenitor cells [71]. This has been attributed, in part, to decreased proliferative capacity of the luminal alveolar progenitor in the absence of CRIPTO [45,72]. Interestingly, neither WAP nor Mouse Mammary Tumor Virus (MMTV) promoter-driven CRIPTO overexpression led to appreciable morphologic or histological changes the mammary gland of nulliparous mice prior to 10 weeks of age, even though MMTV drives recombination in utero, suggesting that production of CRIPTO within the developing mammary epithelium at all time points prior to alveologenesis may be dispensable [71]. However, CRIPTO overexpression during development enhances migratory and branching morphogenesis potential of mouse mammary epithelial cells in vivo and in an in vitro wound healing/regenerative response assay [73]. Furthermore, treatment of fetal mammary stem cells (fMaSC) ex vivo with exogenous CRIPTO increases the relative proportion of cells co-expressing Krt8 and Krt14, luminal and basal lineage markers, respectively. Lineage marker co-expression is a hallmark of plastic uncommitted cells in normal and neoplastic tissue [74,75,76]. Functionally, this presents as improved serial passage potential and increased transplantation take rates of cells orthotopically implanted into cleared mammary fat pads—two measures of mammary stem cell function [42]. In this setting, surface localization of glucose regulated protein, 78kD (GRP78), a requisite CRIPTO surface binding partner, specifically marks a stem-like population that accordingly shows increased ability to transplant and is responsive to exogenous CRIPTO [42]. It is unclear whether this population is entirely distinct or partially overlapping with the progenitor population influenced by CRIPTO during alveologenesis in vivo. Another potential explanation for differing CRIPTO responsiveness is that different stages of development rely on CRIPTO from different sources and that early development may supply sufficient CRIPTO in trans. Indeed, during early mammary development, CRIPTO expression is greatest in the fat pad surrounding the mammary rudiment [42]. Models targeting the appropriate stromal cell or sequestering soluble CRIPTO in the extracellular space would be needed to formally test this possibility.

### 2.3. CRIPTO Re-Emerges in Wound Healing

There is mounting evidence indicating that part of CRIPTO’s elusiveness involves its relegation to allostatic processes during injury and regeneration that can call it out from ‘hiding’. In zebrafish fin regeneration studies, CRIPTO was found to be induced by glucocorticoids, in a manner that is conserved in mammals, while retinoic acid (RA) inhibited CRIPTO induction in this setting [77]. In a mouse model of acute injury, secreted CRIPTO promotes myogenic commitment and differentiation, enhances early regeneration and reduces necrotic fibrotic areas [37,78]. CRIPTO is also increased in immune cells upon infiltration of injured muscle. Myeloid-specific CRIPTO deletion hampers conversion of infiltrating macrophages towards anti-inflammatory macrophages, shifting the balance in favor of endothelial-to-mesenchymal transition, rather than stimulating regeneration [47]. CRIPTO’s pro-proliferative and regenerative effects in muscle satellite stem cells are mediated via interactions with MYOSIN II and ROCK1/2 kinases, mechanical stress response proteins that also seem to control the subcellular localization of CRIPTO [79,80]. Very recent studies of osteo-arthritis in mice propose a role for CRIPTO in enhancing chondrocyte proliferation by forming a complex with TGF-β and its type I receptor ALK1 and increasing Smad1/5 signaling [38]. In pancreatic regeneration studies, CRIPTO is predominantly found in smaller ducts that are mutually distinct from differentiated insulin-positive pancreatic cells, which are characterized by high levels of ACTIVIN A signaling, while its expression in non-regenerating pancreatic tissue is scarce and found only in blood vessels [36]. Blocking CRIPTO also drives dopaminergic neuron over astrocyte differentiation during neural differentiation from embryonic stem cells, thus implicating CRIPTO in control of neural stem cells and lineage fate decisions and making it an important consideration in the development of bioscaffold based stem cell transplantation approaches for neural regeneration following injury [81,82]. In a hindlimb ischemic model, CRIPTO was shown to interact with the concomitantly induced and overexpressed GRP78 and to synergistically increase human mesenchymal stem cell (MSC) proliferation, migration and invasion potential with potential applications for MSC-based transplantation and regeneration approaches for treating wounds [83]. There is an interesting parallel here between the hypoxic microenvironment associated with this ischemia model and the role of hypoxia in CRIPTO-mediated hematopoietic stem cell maintenance, however here as exactly how stress signals are integrated across multiple cell types has not been investigated thoroughly, and, although CRIPTO is a known Hif1 α target [84], it remains to be determined if Hif1 α is required for all adaptive responses that go through CRIPTO. 

As a response to various types of wounds CRIPTO may also be a key regulator of inflammation. CRIPTO is an early response gene during neuroinvasion induced by human immunodeficiency viruses in the cerebral cortex, along with immunomodulatory genes. In this setting CRIPTO expressed in CNS may play a neuroprotective, survival role [48]. Moreover, treatment of CD4+ T cells with soluble CRIPTO upregulates proinflammatory TNFα expression, as well as interleukin-4, interleukin-6, and ONCOSTATIN M [85]. CRIPTO enhances macrophage phagocytic activity and upregulates the production of anti- and pro-inflammatory cytokines via the NF-κB signaling pathway; interacting proteins include members of actomyosin, stress response, and exosome pathways [80,86]. Synergy between CRIPTO and NF-κB signaling has also been reported in HCC [87]. Although inflammation is part of the normative process of wound healing, chronic inflammation and resultant fibrosis can promote cancer.

### 2.4. A Role for CRIPTO in Fibrosis

In the development of cardiac tissue, CRIPTO is predominantly expressed during early stages within the ventricles and outflow tract and like other tissues it decreases upon reaching homeostasis [46]. Accordingly, CRIPTO, as well as its potential cooperating factors WNT/β-CATENIN, TGF-β family and bone morphogenetic proteins (BMP) have been characterized as essential proteins for heart induction [84,88,89,90]. Deletion of CRIPTO in ESCs yields cardiomyocytes with contractile fibers but these fail to beat [91]. CRIPTO was shown to act through the G-protein-coupled receptor APJ and its ligand APELIN to specify ESC toward the cardiac lineage through extracellular signal-regulated kinase/p70S6 kinase signaling pathways [92]. APELIN has also been identified as an important regulator of cardiovascular homeostasis and hypoxia-induced angiogenesis in vivo [93]. Here again, this time in ESCs, hypoxia is reported to drive expression of CRIPTO and the presence of both CRIPTO and hypoxic conditions are necessary to promote complete differentiation of ESCs into cardiomyocytes, further underscoring the likely dependency of CRIPTO on specific contextual cues to be fully functional [84]. 

As in most tissues, CRIPTO expression in adult heart is negligible. However, we (M.KdJ) and our collaborators recently reported that injury models in both the heart and liver lead to CRIPTO upregulation [35]. The expression of CRIPTO in the heart shortly after experimental myocardial infarction (MI) concomitant with, and even preceding, the appearance of myofibroblasts (MFBs), suggests an activating role for CRIPTO in cardiac fibrosis [35,84]. In fibrotic cardiac tissue only a very limited amount of CRIPTO was observed, whereas in MI, pressure overload and ex vivo mouse models of cardiac fibrosis, CRIPTO was upregulated. The difference can likely be explained by the dynamic expression of CRIPTO during the different stages of fibrosis. In a recent study CRIPTO was detected in blood samples of patients with planned MI within 1 h of infarction [87]. Similarly, induction of CRIPTO expression within two hours following ischemic cardiac damage is observed in a porcine model of cardiac injury [84]. In human cardiac fibroblasts pro-survival factor NEUREGULIN-1 has been shown to increase the production of CRIPTO along with inflammatory cytokines INF-γ and IL-1α [94], however whether CRIPTO regulates the cellular function of these fibroblasts or targets other cardiac cell types is not entirely clear.

In healthy hepatic tissues, CRIPTO has been reported to be expressed at low levels [95]. However, CRIPTO has been detected in settings where the high regenerative capacity of hepatic tissue is activated in a dysregulated manner. For instance, high CRIPTO levels have been reported in hepatocellular carcinoma (HCC) tissue [41,87,96] but also in patients with hepatitis B-induced HCC [95]. CRIPTO has also been shown to foster cell proliferation, migration and invasion in HCC, where Lo and colleagues associated it to stemness and poorer disease-free survival by exploring its role in stabilizing Dishevelled-3 (DVL3) and activating WNT/β-CATENIN pathway [97]. The mechanisms of action for this phenotype have been related to the biological function of NANOG which activates NODAL and CRIPTO to promote SMAD3 phosphorylation and *Snail* expression in HCC metastasis [98]. In a non-carcinoma setting, serum CRIPTO levels were also higher in cirrhosis and chronic hepatitis than in volunteer control samples [95]. CRIPTO levels are also higher in liver tissue samples of patients suffering from alcoholic liver disease (ALD) or viral- induced liver cirrhosis compared to control group, while CRIPTO levels in plasma decrease after liver transplantation (LT) in paired pre- and post-LT samples [35]. The association of elevated CRIPTO and disease associated fibrosis in these settings contrasts somewhat with CRIPTO’s amelioration of fibrosis in the myogenic models described above [37,78].

In a murine model of liver cirrhosis induced by repetitive exposure to the hepatotoxin CCl_4_, CRIPTO levels were increased compared to the control group and found mainly in the epithelial compartment [35]. To study the kinetics, dynamics, and stimulus of the reactivated CRIPTO expression, we (M.KdJ) used an acute liver fibrogenesis model in which mice were subjected to a single shot of CCl_4_ followed by liver tissue analysis at various time points. CRIPTO was one of the early response genes to be activated within hours of tissue injury and preceding the appearance of activated MFBs [35]. At the cellular level, it is generally accepted that the key mediators of tissue fibrosis are activated collagen-secreting MFBs. The origin of MFBs is usually tissue dependent, and MFBs have been shown to originate from tissue-resident mesenchymal, epithelial, and endothelial cells as well as from circulating bone marrow–derived fibrocytes [99]. Using reporter assays, CRIPTO was identified to be an upstream direct regulator of αSMA, a marker of transdifferentiated MFBs. The absence of CRIPTO in adult liver and the short reactivation of its expression in a wound healing response may indicate that it plays an orchestrating role in the regulation of tissue injury/inflammation and fibrogenesis. However, the molecular activating stimulus of CRIPTO expression has not yet been elucidated. In another study, in vivo adenoviral-mediated CRIPTO overexpression resulted in higher levels of fibrotic markers, whereas inhibition of CRIPTO by the ALK4^L75A^-Fc ligand trap [42,100], led to improved hepatocyte proliferation and significantly reduced fibrosis, implying a role for soluble CRIPTO. Collectively, these observations indicate that elevated CRIPTO is likely not a simple consequence of the tissue damage or fibrosis, but rather a direct regulator of the cascade of fibrosis by inducing MFB recruitment [35].

**Figure 2:**
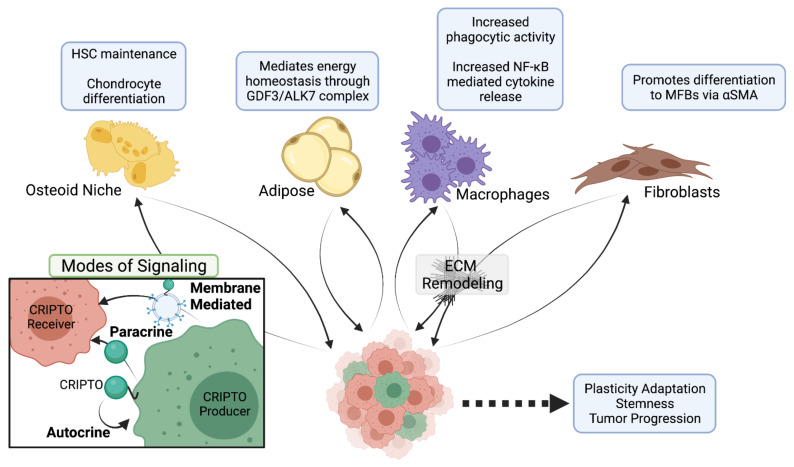
Effects of heterotypic CRIPTO signaling on various cell populations. Created in BioRender 15 August 2021.

Together, these studies support the concept that CRIPTO may remain ‘hidden’ in healthy tissues through the spatiotemporal rarity of its action—only operating in select cells and under select conditions (Figure 2). Importantly, CRIPTO’s involvement in the generation and maintenance of stem cell phenotypes, inflammation, fibrosis and stress adaptation heavily implicate it in the generation and progression of diverse cancers as each of these activities has been widely implicated in cancer initiation and progression.

## 3. Re-Emergence of CRIPTO

### 3.1. Diverse Tumors Invoke CRIPTO

Due to the cellular and contextual selectivity described above, CRIPTO levels are largely negligible at the tissue level in the adult. However, CRIPTO levels are upregulated in an impressive variety of cancers including prostate, hepatocellular carcinoma, pancreatic, bladder, colon, breast, lung, and gastric cancer (Table 1) [53,64,65,96,101,102,103]. CRIPTO is expressed in a high percentage of infiltrating breast cancers with nearly 80% of carcinomas staining positive in a panel of 100 tumors, suggesting its involvement across multiple breast cancer subtypes including hormone receptor positive (ER+PR+), Her2+ and triple negative breast cancer (TNBC) [45]. It is also likely to be functionally involved as overexpression of CRIPTO in the murine mammary epithelium leads to hyperplasia of the ductal networks. However, CRIPTO overexpressing mice are slow to develop mammary adenocarcinomas, and do so at a low penetrance, suggesting that while CRIPTO promotes hyperplastic growth, additional factors or cellular cues are required for progression to frank neoplastic lesions [104]. Similarly, in prostate cancer high CRIPTO expression correlates with poor outcomes [51,53]. CRIPTO expression is predominantly restricted to a stem-like subpopulation of prostate tumor cells and CRIPTO-knockdown reduces the metastatic capacity of these cells, suggesting that CRIPTO plays a role in tumor progression [53]. Furthermore, most analysis of human cancers has obviously focused on established lesions, and consequently, the degree to which CRIPTO marks and contributes to pre-neoplastic states that lead to cancer, remains to be determined.

Despite increased protein levels and in vivo data supporting the role of CRIPTO in tumorigenesis and progression, expression of CRIPTO has been particularly difficult to detect at the RNA level in both bulk and single-cell RNA-sequencing tumor data sets. Although CRIPTO expressed from the *TDGF1* gene is predominant in ESCs and germ cell tumors, a highly homologous pseudogene, *TDGF1P3*, is expressed and likely produces functional protein (CRIPTO-3; CR-3; TDGF3) in various cancer types, including colon, breast and lung [105,106]. This raises the possibility that CRIPTO-3, not CRIPTO (i.e., CR-1), is responsible for the associated tumorigenic effects. Despite high levels of CRIPTO-3 message identified by RT-qPCR, relative levels remain low in single-cell RNA-seq data sets. As a pseudogene, CRIPTO-3 message may lack a poly(A) tail in which case traditional RNA-seq libraries generated by poly(A) enrichment would fail to identify it [107]. CRIPTO is also known to undergo a variety of post-translational modifications, with one study showing that a truncated version of CRIPTO is expressed in the majority of colorectal neoplastic lesions [108]. However, the functional significance of this truncation is unknown. Understanding, CRIPTO pathway activation at single cell resolution may be essential to understanding how and where it works in tissue development and tumorigenesis but may require the use of alternative analytical techniques more capable of detecting and distinguishing family members and isoforms than those currently employed.

### 3.2. CRIPTO in Specialized Tumor Cells

Several studies have demonstrated that subsets of tumor cells across many tumor types exhibit enhanced tumor propagating capacity, leading to their receipt of the moniker, ‘cancer stem cells’ [109,110]. The strictness with which the cells of various tumors recapitulate a stem -> transit amplifying -> progenitor -> differentiated cell hierarchy is a matter of debate and likely varies significantly by tumor type [1,111]. Nevertheless, morphological and functional heterogeneity is a well-established and clinically challenging feature of most tumors and mounting evidence indicates that CRIPTO could be of particular importance in determining the phenotypes of specific subsets of tumor cells.

Even in broadly “stem-like” germ cell tumors there is heterogeneity of CRIPTO expression. For instance, the embryonal carcinoma cell line NTERA2 contains both CRIPTO-high and CRIPTO-low cells, which are associated with high and low tumorigenic capacities respectively [112]. This same CRIPTO-high population shared gene signatures with embryonic stem cells including increased expression of *Sox-2*, *Oct-4* and *Nanog* [112]. Differential expression and localization of CRIPTO binding partners would be expected to amplify discrete areas of CRIPTO signaling as GRP78 is necessary for MaSC activity and are responsive exogenous CRIPTO [42]. This suggests a model where CRIPTO’s feed forward role in regulating ESCs during development may extend to certain stem-like populations in tumors, promoting their emergence, persistence and/or aggressiveness. Similarly, in a glioma cell line, exogenous CRIPTO upregulates endogenous CRIPTO expression in a sub-population of cells. This feed-forward loop increases an initially small population of CRIPTO-positive cells, highlighting a mechanism by which a small portion of CRIPTO-positive cells can have a large impact in the right environment [113]. In vivo the cellular heterogeneity could reflect the heterogenous tumor landscape, where specific tumor cell subpopulations are responsive to different microenvironmental cues and varied heterotypic signaling.

CRIPTO has been identified as a marker of CSCs in several cancer models including prostate, melanoma, colon, esophageal and hepatocellular carcinoma (HCC) [69,114,115,116]. The biological effects of CRIPTO within these CSC compartments remain uncertain. Silencing of CRIPTO in colorectal CSCs reduced the proliferative capacity of this population [69]. Somewhat paradoxically, soluble CRIPTO generated by the introduction of a C-terminal truncating mutation reduced the self-renewal capacity of CSCs derived from murine iPSCs [117]. These seemingly disparate results would suggest that not all CSCs respond to CRIPTO in the same manner. Differences in the surface interactome or other environmental factors such as the available local ligand pools experienced by these cells may account for the observed phenotypes. Alternatively, or in addition, it is possible that *cis* and *trans* CRIPTO signaling regulate CSC populations differently, or truncation of the C-terminus of CRIPTO produces a form that does not recapitulate the biological activity of endogenous CRIPTO.

It is also unclear whether these CRIPTO-positive CSC populations reflect the transformation of a CRIPTO-positive stem cell or niche cell, or alternatively reflect de-differentiation of cells to a stem-like CRIPTO expressing/responsive state. Should these populations represent de-differentiated cell types, the obvious question is what factors reprogram the system to enable CRIPTO reemergence? Recently, we (BTS) showed within MDA-MB-231 xenograft tumors that CRIPTO immunoreactivity localizes to tumor microdomains exhibiting hallmarks of stress such as hypoxia [118]. Although it is known that such tumor microdomains include both xenografted tumor cells and infiltrating host stroma, it is not yet clear which cells are signaling to which other cells through the agency of CRIPTO. One intriguing possibility is that inflammatory infiltrating fibroblasts (and/or possibly macrophages) produce or respond to CRIPTO in the context of necrotic tumor regions and associated detritus or fibrosis.

This would be consistent with our demonstration of CRIPTO production by F4/80 positive (macrophages) and F4/80 negative stromal cells in early mammary development, and recent findings in mouse models of acute muscle injury and Duchenne muscular dystrophy showing the expression of stromal derived CRIPTO (Table 1) [42,47]. Furthermore, hypoxic conditions within these tumor regions may also influence local CRIPTO production as a function of HIF1α binding to hypoxia response elements (HREs) within the CRIPTO promoter [84]. This harkens to the integration of hypoxic stress and CRIPTO signaling in HSCs, ischemia models and cardiomyocytes [39,83,84]. Alternatively, these zones are likely starved for other nutrients and, in this regard, we found that nutrient deprivation in vitro (i.e., serum withdrawal or glycolytic blockade) confers cancer cell CRIPTO dependency [118]. Thus, microenvironmental cues such as hypoxia or metabolic restriction may explain the spatial heterogeneity of CRIPTO expression tumors. Finally, expression of CRIPTO surface binding partners may sequester soluble CRIPTO to specific cellular regions thereby facilitating non-cell autonomous CRIPTO signaling. The effects of CRIPTO in these domains may be further affected by the levels of local growth factors and TGF-β family ligands.

A fraction of GRP78 localizes to the cell surface from the endoplasmic reticulum in the presence of diverse stresses where it binds CRIPTO and facilitates downstream signaling throug c-Src and NODAL [100,119]. Supporting the importance of contextual alterations to the surface proteome, GRP78 and CRIPTO co-localize along acellular regions within xenograft mammary tumors [118]. These acellular regions are characterized by relatively high levels of hypoxia. Subsequent inhibition of CRIPTO in these xenograft tumors reduces proliferative capacity specifically in cells with high levels of HIF1α, supporting the notion that CRIPTO signaling is necessary for tumor growth in hypoxic domains [118]. In vitro proliferation assays of NCCIT embryonal carcinoma cells and mammary tumor cell lines show CRIPTO inhibition does not reduce proliferative capacity under standard culture conditions supplemented with sufficient nutrients, however, in the presence of the inhibitory glucose mimetic, 2-deoxy-D-glucose (2-DG) where CRIPTO inhibition did significantly reduce cellular proliferation, surface localization of GRP78 was increased [118]. This is consistent with a crucial role for cellular stress (in this case metabolic stress) in regulating CRIPTO signaling and responsiveness. It is also consistent with GRP78 as a key stress response component of the pathway since diverse stresses are known to induce GRP78 and trigger its transit to the cell surface [120]. It remains to be determined if CRIPTO activating stresses operate independent or all funnel through a central signaling node such as ER Stress. CRIPTO’s role in neoplastic settings may be a result of a complex regulation of transcriptional regulation and cellular localization of signaling partners. Given the altered signaling capacities of CRIPTO in the presence of surface-localized GRP78, it seems likely that there are unidentified stress-dependent alterations to the CRIPTO interactome. Such changes may exist as a mechanism by which cells are able to undergo reprogramming in response to microenvironmental cues.

These observations all point to CRIPTO as an environmentally sensitive mediator of stem cell phenotypes and suggest its reemergence in cancer may largely reflect expansion of the stem cell phenotype. In this regard, it is unclear whether CRIPTO’s well known effects on EMT are synonymous with its promotion of stemness [121], or whether they represent a separate mechanism by which CRIPTO can promote plasticity and intratumoral heterogeneity to impact tumor progression and treatment resistance.

### 3.3. CRIPTOs Remergence in Cancer May Reflect Tumor Vascularization

As tumors grow, they outstrip the local vasculature capacity and supply of nutrients leading to hypoxic environments and cellular starvation. As in normal injury responses, these conditions can elicit signals for the generation of new vessels. Thus, a reprise of angiogenic roles as described in placental development, where CRIPTO was shown to promote VEGF and NOTCH signaling to ensure proper vasculature formation at the maternal-fetal interface may also underly part of CRIPTO’s reemergence in tumors [32]. Evidence shows that proliferation, migration, and invasion are enhanced by CRIPTO, properties that have also been shown to stimulate tumor angiogenesis [4,6]. Bianco and colleagues showed that treatment of HUVECs with recombinant CRIPTO augments levels of MAPK, AKT and c-Src phosphorylation and induces tube-like structures. Similarly, in vivo experiments showed that overexpression of CRIPTO in MCF-7 breast cancer cell xenografts enhanced tumor neovascularization [122].

Alowaidi and colleagues showed using KEGG pathways database that 47 signaling pathways presented significant changes in protein phosphorylation following CRIPTO stimulation in glioblastoma (GBM) cells. Among them, the most affected pathways were MAPK, FAK and erbB signaling, which are known to regulate cellular functions, such as cell motility, invasion, migration, survival, and inflammation. Interestingly, in this study they pinpointed CRIPTO as a likely mediator of angiogenesis via activation of epithelial growth factor receptor (EGFR) (Ser1070)/Her2 (Tyr877) of the erbB pathway and subsequently c-Jun (Ser63) [123].

Consistently, investigation of ectopic expression of CRIPTO in clear cell renal cell carcinoma (ccRCC) cell lines confirmed its involvement in proliferation, migration, invasion and angiogenesis. In these studies, Caki-2 cells which have relatively low levels CRIPTO expression, were infected with hLV-CRIPTO, resulting in significantly increased proliferative capacity [56]. Cell migration and invasion of Caki-2 cells were assessed using trans-well assays, whereas in vitro angiogenesis tests using a HUVECs tube formation assays showed increased tube formation when HUVEC cells were cultured in conditioned media from Caki-2 cells infected with LV-CRIPTO compared to the control group. CRIPTO effects on in vivo angiogenesis were further investigated using a CAM assay, and CRIPTO’s overexpression was associated with increased vascular neogenesis [56].

Wu et al. showed that CRIPTO downregulation in prostate carcinoma (PCa) led to a dramatic inhibition of cellular migration, invasion, and proliferation, inducing cycle arrest in G1 phase [124]. In this study, CRIPTO siRNA suppressed the secreted level of vascular endothelial growth factor, and reduced protein level of Vascular endothelial growth factor receptor 2, the major mitogenic mediator and permeability enhancing effector of VEGF [124,125]. In regenerative therapy research it has been shown that overexpression of GRP78 along with CRIPTO, enhanced human mesenchymal stem cells (hMSC) proliferation, migration and invasion as well as angiogenesis around transplanted ischemic site via JAK2-STAT3 pathway [83]. Thus, through the induction of soluble angiogenic factors such as VEGF and governance of angiogenic phenotypes (whether acting on endothelial compartment or through vascular mimicry), CRIPTO expression in tumors could reflect tumor associated angiogenesis. It is also tempting to speculate in this regard that hypoxia secondary to tumor cell proliferation is the principal CRIPTO inductive cue.

### 3.4. CRIPTO as a Wound Healing Hallmark and a Fibrotic Marker in Cancer

The concept of tumors being “wounds that do not heal” accurately highlights the similarity of tumor formation to organogenesis and wound healing response. In normal tissues wound healing is tightly regulated and resolves completely, while in tumors this wound response is exacerbated [126,127]. Dysregulated inflammatory responses and aberrant wound healing have long been thought to contribute to carcinogenesis. Inflammatory responses leading to fibrosis are driven by a plethora of initiating factors, including repeated non-homoeostatic conditions, physical/mechanical and chemical insults, radiation, pathogens, autoimmune reactions, and other physiological comorbidities. A classic example is inflammatory liver disease, which is known to predispose to tumorigenic transformation into HCC [128]. In such contexts, the emerging roles for CRIPTO in fibrotic wound healing may explain another reason for its reemergence in cancer.

It is worth noting that several of the pro-inflammatory triggers mentioned above are initiated or aggravated by tumorigenesis or even by tumor therapy. Accordingly, chronic inflammation and subsequent fibrosis are not only linked to increased risk of developing cancer, they often persist in cancers or emerge as a function of tumor growth and progression as well. In the context of breast cancer, for instance, the presence of periductal fibrosis in ductal carcinoma in situ (DCIS) is correlated with an increased risk of progression to invasive breast cancer [129]. Similarly, fibrogenic cytokines, including IL-6, TGF-β and PDGF are expressed in melanomas but not in benign nevi [130]. From a biophysical perspective, fibrotic remodeling of the ECM by cancer associated fibroblasts (CAFs) is required for tumors to reach a point of ‘tensional homeostasis’ that is uniquely tuned to specific cancer types [131]. Within tumor types that require rigid ECMs, including breast and pancreatic cancer, ECM density produced by CAFs is associated with poor prognosis and aggressive subtypes [132,133].

In addition to altering the surrounding microenvironment to suit established tumor cells, CAFs can influence cellular reprograming of healthy tissue. In vitro co-culture of a benign hypertrophic prostate cell line, BPH-1, with CAFs was sufficient to induce a malignant transformation and promote tumor formation in vivo [134]. In HCC, CAFs increased the self-renewal capacity and tumorgenicity of CD24+ tumor cells via HGF and IL-6 [135]. The crosstalk between CAFs and tumor cells appears to be bidirectional as CAFs and NSCLC cells alter each other’s metabolic profile via a ROS and TGF-β mediated mechanism [136]. Given the established role of CRIPTO in modulating TGF-β signaling and altering fibrosis in homeostatic tissue following insult, it seems likely that CRIPTO also plays a role in regulating fibrosis in neoplastic lesions. Supporting this idea, activated MFBs/stellate cells in pancreatic cancer can induce fibrosis following chronic inflammation and have been reported to influence pancreatic CSCs *in trans* by secreting CRIPTO and NODAL [137,138].

While classically thought of as a single population marked by α-SMA, cancer associated fibroblasts (CAFs) are now accepted to represent a diverse class of cells with various subpopulations conferring either pro- or anti-tumorigenic effects [139]. Different subtypes of breast cancer have been shown to harbor unique CAF subpopulations. CAFs associated with the aggressive triple negative subtype actively immunosuppress the local tumor microenvironment by promoting differentiation of FOXP3+ regulatory T cells [140]. Given the heterogeneity of CRIPTO expression in various tissues, it is interesting to postulate whether CRIPTO expression delineates different classes of CAFs and may explain their tumor-promoting capacity.

## 4. Classical and Emerging Mechanisms of Signaling

### 4.1. CRIPTO Integrates TGF-β and Growth Factor Related Signaling Pathways

As we described at the outset of this review, much is known about molecular mechanisms of CRIPTO signaling especially as it pertains to TGF-β pathway modulation (see Klauzinska et al. [3] for a review), however our knowledge of the mechanisms that connects tumor associated pressures to CRIPTO regulation, signaling and cancer-dependent cellular outputs remains insufficient. In addition to its role in TGF-β signaling, CRIPTO has a well-documented but poorly defined role in augmenting growth factor signaling and activating c-src/ERK/MAPK cascades and has also been reported to interact directly or indirectly with a variety of key governors of growth and differentiation including NOTCH, WNT and ERBB pathways (Figure 1) (see also [3]). As we (PCG) showed and described above, CRIPTO signaling is also dependent on interactions with surface localized GRP78 (a.k.a. HSPA5, Bip), an endoplasmic reticulum (ER) chaperone protein and central regulator of the unfolded protein response, that re-localizes to the cell surface under diverse cellular stresses [141]. Given this nexus, CRIPTO likely orchestrates a delicate balance between these complex signaling programs and cellular stresses, responding to contextual cues to regulate embryo-critical cellular phenotypes that can also be hijacked by cancer cells to promote tumor progression. Precisely how this balance manifests tumor prone phenotypes and the molecular details of its ‘rekindling’ remain to be uncovered.

### 4.2. Regulation of CRIPTO Signaling

CRIPTO is regulated at transcriptional, translational and post-translational stages, but little understanding exists regarding how regulation at these various stages relate to its cellular and tissue level effects. In general, it appears that transcription factors related to stemness upregulate CRIPTO expression while pro-differentiation factors repress it. As previously discussed, CRIPTO is subjected to differing transcriptional regulatory processes including feed-forward loops of transcription factors important in early developmental processes including NANOG and OCT4 [29,112]. Additionally, CRIPTO was identified as a target of WNT (another oft touted stem cell factor) described in colorectal carcinoma and intestinal stem cells [68,69]. The CRIPTO/WNT program is likely also borrowed from the ontological repertoire as canonical WNT/β-CATENIN regulation of CRIPTO expression has also been demonstrated during early embryonic development and plays a role in CRIPTO’s ability to regulate anterior posterior axis patterning [142]. The CRIPTO promoter and first intron contains three TCF binding elements that are regulated by the canonical WNT/β-CATENIN signaling pathway [143]. CRIPTO may act in a feed-forward WNT-signaling loop since CRIPTO can amplify WNT/β-CATENIN signaling by binding directly to the WNT co-receptors LRP5 and LRP6 and facilitate WNT binding to FZD receptors [143,144]. CRIPTO has also been proposed to regulate EMT and stemness via stabilizing the WNT pathway regulator disheveled segment polarity protein 3 (DVL3) in several tumor types including prostate cancer and hepatocellular carcinoma [97,145]. Further examples of CRIPTO transcriptional control that govern self-renewal vs. differentiation include the action of both RA and germ cell nuclear factor (GCNF), which promote differentiation processes and have been shown to repress CRIPTO expression. While GCNF can directly bind to a DR0 element within the CRIPTO promoter, it is unknown whether RA suppresses CRIPTO expression directly [106,146]. Liver receptor homologue-1 (LRH) is important in maintaining *Oct-4* expression in ESCs and competes with GCNF for the same DR0 element within the CRIPTO promoter [146].

In neoplastic settings, transcriptional regulators of CRIPTO do not necessarily represent discrete tumor suppressing or tumor promoting phenotypes. Many dynamic tissues in mammals are under hormonal control that gets exploited in oncogenesis. Hormone receptors may be key regulators of CRIPTO function as well. For instance, CRIPTO expression can also be upregulated through progesterone receptor (PR) signaling [71]. In the mammary gland progesterone is essential for maintaining normal mammary stem cell and cancer stem cell populations, however, in endometrial cancer progesterone is generally considered tumor suppressive [147,148,149]. In mammary cells, CRIPTO and PR appear to exist in a positive feedback loop in which CRIPTO and PR are sufficient to induce expression of each other (though mechanisms remain to be clarified), implying that hormone fluctuations in sensitive tissues may impact CRIPTO levels and activity [71]. Classical reciprocal transplant experiments showed stromal PR expression is necessary for ductal development while epithelial PR expression is required for lobuloalveolar development [147]. Thus, both the mammary stroma and epithelium express PR, however they have differing respective impacts on mammary development that may be recapitulated in different types of cancer. These differing compartmental roles for PR may inform CRIPTO’s variable spatiotemporal expression pattern and epithelial knockout phenotypes. In breast cancer, progesterone signaling is known to be a mitogenic agent and induce a pro-survival phenotype in recipient cells [150]. PR expression also contributes to major molecular phenotype descriptions in breast cancer, though the potential subtype specific effects of CRIPTO blockade as a breast cancer therapy are underexplored.

Similarly, androgen receptor (AR) signaling represents the dominant route supporting prostate cancer cells proliferation. Previous studies have shown that CRIPTO is expressed in AR positive and negative cellular models and CRIPTO knockdown resulted in lower proliferation levels in both settings [53,114]. It was reported that AR negative PCa models such as Du145 and PC3 express stable levels of NODAL while it was not detected in AR positive LNCaP [151]. Importantly all cell lines expressed NODALS’s co-receptor, CRIPTO, but lacked LEFTY, a critical negative regulator of NODAL signaling. Recombinant human NODAL triggered downstream SMAD2 phosphorylation in DU145 and LNCaP cells and attenuated AR signaling, suggesting that CRIPTO might mechanistically support the acquisition of a castration resistant phenotypes [151]. Paradoxically, in the zebrafish injury model, glucocorticoid receptor (GR) signaling induces CRIPTO expression but inhibits fin regeneration [77]. In cancer GR agonists are often tumor suppressive, likely because of downregulation of hormone signaling. However, GR signaling can become tumor promoting upon the loss of hormone receptors [152]. In cancers, CRIPTO expression may reflect aberrant developmental signaling associated with the stem cell promoting and differentiation inhibiting transcription factors. This process is likely highly context specific as these factors drive complex networks rather than single oncogenes.

In light of the recent implication of CRIPTO in fibrosis as described above, it is interesting to consider how different ECM substrates (these could include e.g., specific signaling domains and varying stiffness) may lead to different levels of CRIPTO expression induction in colon cancer cells [153,154]. Specifically, heparin proteoglycan induces autocrine CRIPTO expression in metastatic colon cells when grown in fibronectin and plastic conditions and decreases CRIPTO mRNA expression in cells plated in collagen type I and IV [153,154]. The fact that hepatocyte-derived ECM from the liver, which is a common metastatic site for colon cancer, stimulates CRIPTO expression in highly metastatic colon cancer cell line KM12SM may indicate a selective advantage for CRIPTO-expressing colon cancer cells to metastasize to the liver [153,155]. The likely interconnection of CRIPTO function with ECM proteins, is highlighted by its association with integrin signaling [156] and its binding to Glypican 1, a heparin sulfate-proteoglycan, leading to the downstream activation of c-Src kinase and EMT promotion in mammary cells [157].

CRIPTO may also be susceptible to post-transcriptional regulation involving microRNAs, although this has not been studied extensively. miR-15b and miR-205 have been inversely correlated with CRIPTO expression in gliomas and non-small cell lung cancer (NSCLC) respectively [158,159]. However, only miR-15b has been shown to reduce CRIPTO expression and subsequently reduce proliferation in glioma cell lines [158]. Interestingly, miR-205 regulates neuropilin-1 (*Nrp1*) expression in uveal melanoma and CRIPTO has been implicated in regulation of *Nrp1* however, this connection remains tenuous [123,160]. CRIPTO has also been shown to regulate expression of Zeb (*Zeb1*) and Vimentin (*Vim*) through downregulation of miR-205 [161]. However, the mechanisms by which CRIPTO and miRNAs function together remains an understudied area in the field.

Post-translational modification of CRIPTO is almost wholly un-studied. And, it has never been determined how much (quantitatively) CRIPTO is required for adaptive responses in normal or tumor tissues, much less how much CRIPTO activity is needed. Functionally important levels of CRIPTO in non-germ cells cancers appear to be orders of magnitude lower than seen in germ cell tumors such as NTERA and NCCIT. This may simply reflect the proportion of the cells within the tumor that are in the stem cell state. Alternatively, this may be due to expression of different isoforms of CRIPTO and the ability to detect these variants with current sequencing platforms as previously discussed. Fully translated CRIPTO undergoes significant post-translational modification that can affect its subcellular localization and subsequent signaling. Following translation, CRIPTO is cleaved after proline 51 prior to additional modifications [162]. Subsequent glycosylation events occur in the trans Golgi network (TGN). However, blocking of ER to TGN transport with brefeldin A did not disrupt membrane localization of CRIPTO despite inhibiting CRIPTO secretion [163]. This suggests trafficking through the TGN is necessary for generation of soluble CRIPTO. In our hands collectively, recombinant CRIPTO often fails to recapitulate CRIPTO phenotypes which may be a result of improper glycosylation. Recently soluble CRIPTO produced from 293T cells was shown to be sufficient to rescue gastruloid formation in CRIPTO knockout 3D cultures [164]. Furthermore, recombinant CRIPTO is sufficient to rescue *Oep* knockout in zebrafish although mutation of Asn63, a N-glycosylation site, reduced the efficacy of this rescue, suggesting that recombinant CRIPTO may replicate endogenous levels in certain contexts and this may be regulated in part by post-translational modifications [162]. Future work is needed to understand the contributions of different post-translational modifications on CRIPTO function and stability.

Thus, CRIPTO signaling and that of its partners may be highly context dependent reading out nutrient availability alongside the distribution of different hormones, cytokines, TGF-β and other growth factors that can regulate CRIPTO expression, post-translational modification and/or shedding [165].

### 4.3. CRIPTO Signaling in Trans

In addition to post-translation modifications in the TGN, generation of soluble CRIPTO has been reported to require the presence of PGAP6 to cleave GPI-anchored CRIPTO and generate lyso-CRIPTO that is released from the cell when the lysophosphatidylinositol is removed by phospholipase-D. PGAP6 appears to be highly specific in its ligands as it does not generate soluble Cryptic despite the high homology between CRIPTO and Cryptic [166]. While soluble CRIPTO generated by PGAP6 can signal through NODAL *in trans*, other work has shown that C-terminal CRIPTO mutants lacking a GPI anchor are unable to induce NODAL signaling [167]. This raises the question of the biological relevancy of C-terminal CRIPTO mutants that are widely used in the field.

Soluble CRIPTO has been shown to signal *in trans* extensively through canonical CRIPTO pathways including NODAL/ACTIVIN and c-Src [18,157]. Additionally, CRIPTO is capable of mediating heterotypic signaling between stromal and parenchymal tissue. Overexpression of CRIPTO in LO2 hepatocytes is sufficient to induce expression of IL-10, TNFα, IL-6 and IL-1β in proximal macrophages via NFαB signaling due to IαB phosphorylation and p63 nuclear translocation [86]. CRIPTO-positive cells appear in cirrhotic livers and precede the emergence of stellate cells in murine models of chronic fibrosis. Moreover, overexpression of CRIPTO in the liver is sufficient to induce stellate cell formation and this effect is further enhanced in an acute murine model of liver fibrosis [35]. This parenchyma-stroma axis appears to be bidirectional as we (BTS) have also shown 3T3 fibroblasts produce CRIPTO and can augment colony formation in mammary stem cells in an in vitro co-culture model and this effect is ameliorated upon genetic and pharmacological silencing of CRIPTO [42].

While CRIPTO can signal *in trans* upon cleavage of its GPI anchor, there are contextual circumstances where secreted CRIPTO may maintain and require the GPI anchor to remain membrane-attached for biological activity [167]. However, this does not exclude the possibility that non-cell autonomous CRIPTO signaling is mediated predominantly by a membrane-bound form of CRIPTO. Indeed, membrane bound CRIPTO can be transferred intercellularly and alters the physiochemical structure of the plasma membrane [168]. In addition, proteomics of Flag IPs from MCF10a CRIPTO-Flag overexpressing cells reveal an enrichment of proteins related to extracellular vesicles [80]. Extracellular vesicles can be comprised of apoptotic bodies, microvesicles and exosomes which are formed by programmed cell death, outward membrane budding and formation of multivesicular endosomes respectively. CRIPTO has been implicated in shuttling proteins (specifically Nodal) to early endosomes, which is necessary for canonical TGF-β signaling through SMAD2/3 as mediated by the endosomal-membrane bound protein SARA [169]. Moreover, CRIPTO-marked endosomes are characterized by high levels of flotillin, a widely accepted marker of extracellular vesicles that regulates extracellular vesicle cargo [170]. Together these associations raise the possibility that subcellular localization of CRIPTO (e.g., in lipid rafts, endosomes and exosomes) may be key to its signaling including especially activation of c-src and integration with regulated compositions of local, subcellular proteomes [165,167]. Sorting and trafficking mechanisms regulating CRIPTO or regulated by CRIPTO could highlight a role for CRIPTO signaling beyond its traditional role as a modulator of SMAD2/3. Indeed, CRIPTO also facilitates growth factor-like signaling from such diverse pathways incorporating AKT, c-Src, WNT, NOTCH and ERBB [123,157,171], that a central trafficking role could provide a reasonable explanation for its apparent pleiotropy. Although, TGF-β signaling could be similarly regulated by such a trafficking role, CRIPTO can regulate anterior-posterior axis patterning independent of the NODAL-SMAD2/3 signaling axis and its less well explained growth factor signaling cascades may be of critical importance in these effects [172]. Consistent with this, in cancer, we have shown inhibition of CRIPTO with a soluble inhibitor, Alk4^L75A^-Fc, reduces primary and metastatic tumor burden in a breast cancer xenograft model. This occurs despite the fact Alk4^L75A^-Fc did not appreciably disrupt CRIPTO-NODAL signaling in *cis* [118].

## 5. CRIPTO’s Role in Cancer

### 5.1. CRIPTO as a Driver of Tumor Progression and Metastasis

CRIPTO has been implicated in multiple neoplastic settings (Table 1), but how it orchestrates tumorigenesis and tumor progression remains unclear. As previously discussed, CRIPTO overexpression alone in the mammary gland is not sufficient to drive tumorigenesis [104]. However, CRIPTO signaling blockade in MDA-MB-231 mammary tumor xenografts attenuates primary tumor burden, although inhibition of CRIPTO is not sufficient to cause regression [118]. Similar effects were observed in a metastatic mouse model using CRISPR-Cas9 mediated knockout of CRIPTO in the TNBC-like JygMC(A) cell line [101]. Knockdown of CRIPTO in a prostate cancer murine model also severely attenuated tumor growth [53]. Importantly each of these studies showed profound effects on tumor progression and metastasis [53,101,118]. However, in most cases it has not been definitively determined which CRIPTO-regulated cellular phenotypes are responsible for the effect.

CRIPTO’s ability to propagate stem cell-like phenotypes and its association (along with GRP78) in marking and regulating stem cell function implicate cancer stem cell activity in its tumor promoting effects. CSCs may exist as either a tumor initiating population that can differentiate to varying degrees to form a heterogenous population or represent a de-differentiated established tumor cell capable of adapting to new microenvironmental cues and ultimately surviving to seed distant metastatic sites. CRIPTO expression in colonic CSCs appears to be a necessary driver for both primary tumor growth and metastatic disease in a colorectal murine xenograft model [69]. Conversely, mCr-1 vaccination reduced the tumorigenic capacity of CSCs derived from spheroid-cultured murine Her2-neu mammary TUBO cells in vivo [173]. These are just two functional examples of Critpo regulating cancer stem cells from an expanding number of studies which implicate it [174].

Although EMT and CSC identities are likely only partly overlapping [121], promotion of EMT is a well-established CRIPTO signaling output that almost certainly contributes to cancer promotion by CRIPTO. Because several stages in the metastatic cascade are thought to be promoted by mesenchymal phenotypes, CRIPTO’s role in specifically promoting metastasis is in line with its function in promoting mesodermal and endodermal migration in early embryonic development by promoting a mesenchymal cell state [16]. For successful metastasis, cells must dissociate from the primary tumor, circulate to distant sites, and acquire the optimal cell-state to adhere and adapt to the local microenvironment in the new tissue. Within tumors, CRIPTO has been shown to promote mesenchymal transcriptional signatures, including loss of *Cdh1* and other epithelial markers and upregulation of *Vim, Smad3*, *Snai1*, *Zeb1/2* and *Twist1/2*, indicative of cellular EMT [116,118,156]. Conversely, CRIPTO knockdown or knockout reduces the migratory and invasive properties of cells derived from prostatic, liver, renal and mammary tumor tissues, suggesting it has broad tissue type-independent functions that support tumorigenesis [53,56,96,118,124]. Zoni and colleagues investigated CRIPTO and GRP78 modulation in the context of highly metastatic human PCa ALDH^high^ cell subpopulation, previously shown to display high bone tropism [53]. In the context of PCa bone metastasis, CRIPTO was shown to modulate TGF-β signaling, which is one of the key molecules of bone remodeling following metastatic progression and it was previously demonstrated that CRIPTO binds TGF-β and inhibits its signaling, potentially supporting an inhibition of the cytostatic effect of TGF-β in cancer [18,151,175]. Both zebrafish and a preclinical mouse model of bone metastasis have been used to study the role of CRIPTO and GRP78 in aggressive, metastatic human PCa cells. The authors demonstrated that when CRIPTO or GRP78 are downregulated the extravasation potential of stem/progenitor-like cells is inhibited [53]. Chen and colleagues, genetically traced single-cell dynamics of human PCa cells at the early stage of metastasis in a model of zebrafish. Demonstrating that androgen independent PCa cells were able to extravasate from the caudal vein, invade the neighboring tissue, proliferate, and form metastasis around caudal hematopoietic tissue (CHT) in four days. Furthermore, they confirmed that CRIPTO is required to increase mesenchymal potential of cells because by downregulating it they observed a significant reduction in vimentin/E-cadherin ratio in engrafted cells [116]. Consistent with this idea, lung metastasis formation was significantly affected when CRIPTO knockdown cells were injected into nude mice via the tail vein in a model of renal cell carcinoma [56]. The exact mechanism by which CRIPTO regulates EMT is almost certainly multifactorial as CRIPTO is implicated in multiple signaling pathways, including canonical TGF-β, WNT/β-CATENIN, NOTCH and SRC, that are also known to regulate expression of mesenchymal transcriptional signatures [20,145,157]. Whether CRIPTO promotes EMT in multiple subpopulations within a tumor or a single population is unknown. In either case, reversing CRIPTO may mediated EMT induction may be especially advantageous as mesenchymal phenotypes appear to contribute to multiple steps in tumor cell dissemination as well as therapy resistance [66,176].

Of note, the metastatic process often requires the ability of cells not only to acquire an initial mesenchymal state but also subsequently to revert this state and readopt a more epithelial state through a mesenchymal to epithelial transition (MET) [177]. How CRIPTO might be controlled to permit this latter MET process is not well explored. One mechanism may involve suppression of CRIPTO in metastatic sites epigenetically or post transcriptionally. The secreted neuronal guidance protein, NETRIN-1, has been shown to increase expression MET markers involved in cellular adhesion, including E-CADHERIN, while decreasing EMT markers such as VIMENTIN in CRIPTO overexpressing cells [115]. Potential paracrine regulation of CRIPTO, for instance to govern this EMT/MET duality, is likely to be multifactorial, reflecting different pools of effectors and differeing receptiveness of tumor cells to CRIPTO pathway activation. For instance, computational modeling suggests CRIPTO significantly alters the dynamics of GRP78 and maintains its presence on the cell surface and subsequent tumorigenic and EMT effects [178,179]. If this is true local environmental cues, such as hypoxia, may operate by altering the cell surface profile of a cell as a function of increasing CRIPTO expression, until these stimuli subside for instance in a well-vascularized, established metastatic site ultimately downregulating CRIPTO and/or cellular receptiveness to CRIPTO. It will be important to determine if one reason CRIPTO often involved but seldom mutated in cancer is a requirement for this kind of dynamic regulation during various stages of cancer progression.

### 5.2. CRIPTO Action at a Distance

Examination of CRIPTO’s role in distant metastatic lesions reveals that CRIPTO inhibition significantly reduces metastatic burden [53,101,116,118]. CRIPTO-antagonistic actions of select BMP proteins may further substantiate this point, for instance following breast cancer colonization in the lung after intravasation following metastasis [180,181], or metastasis to the bone from breast and prostate cancer models in vivo [182,183]. In this respect, BMP-4 can dramatically repress CRIPTO expression while TGF-β1 can induce CRIPTO expression through multiple Smad binding elements (SBEs) in the CRIPTO promoter region [184]. Whether CRIPTO regulates metastasis through its role as an autocrine or juxtacrine/paracrine factor is not fully understood. As a potential example of paracrine function, CRIPTO was shown to be elevated in nasopharyngeal carcinoma with metastasis and its expression was positively correlated with latent membrane protein 1 (LMP1), which acts as a CD40 functional homolog to prevent apoptosis of infected B-lymphocytes, potentially indicating a role of CRIPTO in the modulation of immune response [185]. This hypothesis is supported by independent findings that CRIPTO modulates macrophage cytokine secretion and phagocytic activity via NFκB signaling [86].

Interestingly, CRIPTO has been associated with many proteins implicated in extracellular vesicle biogenesis and transfer, which has in turn been implicated in regulating immune cell function and also pre-metastatic niches development [80,186,187]. Through an exosome mediated mechanism, CRIPTO might promote mesenchymal traits within discrete tumor domains, while simultaneously priming pre-metastatic niches at distant sites through long range vesicle transfer. This model remains purely hypothetical and requires a better understanding of the effects of CRIPTO in modulating non-neoplastic tissue in the context of cancer including putative metastatic destinations in the adult setting. Nevertheless, in the context of secondary metastatic sites, a rapidly growing body of evidence indicates that a recruitment and subversion of non-malignant host cells at future sites of metastasis activate ECM remodeling programs that subsequently facilitate circulating tumor cell colonization [153]. In pancreatic cancer a multicellular mechanism involving activated MFBs/stellate cells has been reported, whereby stellate cells influence pancreatic CSCs *in trans* by secreting CRIPTO and Nodal, and potentially thereby promoting drug resistance [137,138]. Finally, given the previously described role of CRIPTO in regulating wound associated fibrosis it is possible that CRIPTO regulates metastasis to a significant degree through its actions upon stromal fibroblast cells rather than (or in addition to) epithelial tumor cells themselves. The fibrotic environments may provide a foothold for tumor cells to infiltrate, generating either a protective environment within which they can escape immune surveillance until reactivated, or provide cues to switch them to a dormant state. Determining causality in such situations where metastases may arise decades later, as can be the case in BrCa and PCa, has, however, proven to be a major challenge for the field.

### 5.3. Promoting Metastatic Inroads

Indeed, through its impact on stromal cell function, CRIPTO may be able to promote tumor progression through field effects involving ’s tumor several modes of tumor remodeling. For example, blood vessel formation throughout the tumor tissue provides for the delivery of nutrients, oxygen and systemic or local growth factors or cytokines and removal of metabolites such that angiogenesis is closely enmeshed with tumor growth and metastasis [188]. The association of CRIPTO with angiogenesis that we described above may fuel cancer progression not only by feeding nutrients to a growing primary mass but by providing vascular routes for tumor cell dissemination. Our (BTS) recent work suggests that CRIPTO localizes along hypoxic, acellular domains in a murine breast cancer xenograft model [118]. While this appears at odds with CRIPTO’s role in promoting angiogenesis it may reflect differing thresholds of CRIPTO needed for diverse cellular outputs. The levels of CRIPTO needed to promote angiogenesis are not known relative to the levels required to promote EMT and metastasis. Moreover, we cannot yet conclude what degree of hypoxia may dictate CRIPTO’s angiogenic capabilities or its ability to promote a mesenchymal cell state. Alternatively, there may be temporal dependency between hypoxia and stress induced CRIPTO that explain how CRIPTO can promote aggressive stem cell or EMT phenotypes, prior to subsequent invasion of vessels that enable tumor cell escape.

CRIPTO’s role in fibrosis may similarly provide survival and growth promoting cytokine pools while simultaneously providing a physical collagen tract that facilitate tumor cell dissemination. Further, fibrotic alterations to the extracellular matrix (ECM) are well documented to facilitate tumorigenesis by promoting angiogenesis and inhibiting anti-tumor immunogenic responses [139]. Indeed it is estimated that fibrosis driven by chronic inflammation is responsible for upwards of 20% of cancers [189]. Persistent production of CRIPTO resulting from unresolved inflammation and fibrosis could lead to sustained high levels of local and circulating CRIPTO that could in turn promote aberrant cell proliferation and promotion of plastic cellular phenotypes [78,96].

CRIPTO-activated MFBs can remodel the surrounding ECM and lead to the release of growth factors and cytokines into circulation. These effects, in a chronic setting, can drive pro-tumorigenic changes in angiogenesis and cell growth within the affected tissue and when persistent can even generate a pro-metastatic microenvironment capable of supporting the colonization of circulating tumor cells, and activation of resident dormant tumor cells [190,191]. When present in a primary tumor, similar mechanisms of stromal cell recruitment and chronic activation lead to the development of tumor-associated desmoplasia facilitating tumor initiation and progression to metastasis. Similarly, active remodeling at secondary sites can mimic tissue fibrosis mechanisms, albeit at a microscopic scale, and create pre-metastatic sites that enhance tumor cell colonization and growth [186,190].

## 6. CRIPTO in the Clinic

### 6.1. CRIPTO as a Diagnostic Marker

CRIPTO is necessary for tumor progression and metastatic disease in multiple preclinical models [53,118,173]. And detection of CRIPTO levels may have utility as a predictive and/or prognostic marker for tumor status, for instance by assessing the serum levels of shed CRIPTO in various types of cancer [102,192]. In a prospective study of 138 patients with prostate cancer, low CRIPTO expression following radical prostatectomy predicted biochemical-recurrence-free survival [51]. Moreover, the reported association was statistically in line with the predictive power of Gleason scoring, which is the current standard of care, implying CRIPTO detection carries significant clinical utility [51]. A similar association has also been observed in clear cell renal cell carcinoma. The inverse correlation between CRIPTO levels and patient survival was seen in both solid tumor biopsies and serum (AUC-ROC = 0.897; sensitivity = 78.9%; specificity = 100%) [56]. Serum CRIPTO also predicted outcomes in patients with oral squamous cell carcinoma (AUC-ROC = 0.80; sensitivity = 74%; specificity = 78%). Levels decreased following treatment, highlighting a role for serum CRIPTO in monitoring treatment response [193]. Similar prognostic correlations of serum levels of CRIPTO have been found in NSCLC patients [103,194]. The ability to differentiate healthy vs. cancer patients by serum CRIPTO levels alone indicates CRIPTO’s significant potential as a relatively non-invasive blood-based biomarker. Capitalizing on these findings, CRIPTO is now offered as a part of a point of care screening panel for colorectal cancer including CEA, and an undisclosed extracellular matrix protein [195].

Furthermore, high CRIPTO expression in non-small cell lung cancer (NSCLC), as well as in gastric cancer, has been shown to be a predictor of poor prognosis [65,196]. Interestingly, Xu et colleagues showed in their clinical study that the incidence of distant metastasis for stage I NSCLC patients with high CRIPTO expression was significantly higher than that of patients with low CRIPTO expression [65]. Together with downregulated E-cadherin (found in 70% of cases), upregulated CRIPTO was found in 54% of gastric cancer cases, and this was associated with lymph node metastasis, liver metastasis and late TNM stage [196]. Additionally, in gastric cancer a positive association between CRIPTO and STAT3 was reported [62]. Similar findings supported the involvement of CRIPTO as a predictive biomarker for disease recurrence in colorectal cancer, where patients with high CRIPTO expression were statistically susceptible to a recurrence of the disease and showed poorer disease-free survival than those with low expression (cancerous regions vs. marginal non-cancerous regions) [197].

Whether soluble CRIPTO has a functional role in treatment response or simply tracks with tumor burden is still debated. Overexpression of CRIPTO in HCC xenograft models is sufficient to confer resistance to sorafenib, a potent inhibitor of multiple kinases and this effect is reversible with CRIPTO inhibition [96]. In humans, CRIPTO expression in NSCLC correlates with poor prognosis and increased metastatic disease [65]. Additional work has shown CRIPTO correlated with efficacy of 1st/2nd generation EGFR-TKIs resistance in NSCLC and subsequent knockdown of CRIPTO in vitro restored sensitivity to erlotinib in patient-derived primary cells [161]. More recent work showed that despite correlating with primary tumor burden, soluble CRIPTO was unable to confer resistance to Osimertinib, a 3rd generation EGFR-TKI, in NSCLC [194]. It should be noted that soluble CRIPTO in this study was generated using C-terminal CRIPTO mutants, and we cannot exclude the possibility that this alters a portion of CRIPTO’s signaling mechanism such as localization that is not well understood. These data suggest a need to further deconvolute the role of CRIPTO as a therapeutic agent, particularly in the context of combination therapy in treatment-resistant tumors.

### 6.2. Therapeutic Targeting of CRIPTO Signaling

It is generally accepted that TGF-β acts as a multifunctional cytokine and central mediator in the pathogenesis of fibrosis, making it an attractive therapeutic target [19]. However, the pleiotropic and multifunctional effects of TGF-β and its critical role in normal tissue homeostasis, immunity, and cellular decision-making have raised serious concerns about potential side effects that may be caused by systemic TGF-β blockade [198]. In malignancies, the TGF-β pathway is frequently dysregulated and disruption of this pathway can have complex and opposing effects on tumor growth and spread depending on the context. In epithelial cells, TGF-β has cytostatic effects thus acting as a tumor-suppressor signal under homeostatic conditions. However, during later tumor stages TGF-β promotes EMT and metastasis. It is, therefore, critically important to identify additional components within the TGF-β network that can be specifically targeted for therapeutic purposes without the risk of damaging side effects.

CRIPTO has been studied as both a direct and indirect therapeutic target in pre-clinical animal models. New advances in anti-cancer vaccine development have used CRIPTO plasmid DNA vaccination as successful strategy in targeting mCr-1-positive murine mammary carcinoma cells [173]. A similar approach in metastatic melanoma also elicited a protective response against metastasis to the lung [199]. Phase 1 clinical trials have been designed to determine the role of CRIPTO as a targeting agent for an immunoconjugate carrying the microtubule inhibiting maytansinoid derivative, ravtansine [200]. However, nearly 10 years on the results of this early trial have not been released. In addition to using CRIPTO as a targeting mechanism, there have been several attempts to inhibit CRIPTO directly. Mammalian two-hybrid systems have been used to identify possible CRIPTO inhibitors [85,201]. One compound, alantolactone, has been shown to disrupt CRIPTO/ActRIIA binding and was cytotoxic to colonic adenocarcinoma cell lines despite no toxicity noted to normal cells at high doses [201]. Sulforaphane, a natural product found in broccoli, disrupts binding of CRIPTO to GRP78 and Alk4, although no effects on CRIPTO/NODAL binding are observed [202]. Despite not disrupting CRIPTO/NODAL binding, this compound decreases both NODAL and CRIPTO mRNA expression. At the cellular level, sulforaphane decreased tumor-sphere formation in vitro and also reduced primary tumor volume in a TNBC xenograft model, however, these effects were somewhat modest [202], in contrast to the significant reduction in spontaneous lung metastasis observed following CRIPTO knockdown [101].

CRIPTO has also been targeted by inhibitory monoclonal antibodies which decrease primary tumor volume in testicular and colonic xenograft models [21]. Similar effects have been seen in mammary xenografts models. Administration of mCr-1 plasmid DNA generated an immune response against CRIPTO, which subsequently reduced primary tumor volume and significantly impaired metastatic burden [173]. In line with these results, direct targeting of CRIPTO has also been achieved by using a modified version of the Alk4 extracellular domain (L75A) fused to the Fc region of human IgG [42]. This soluble ligand trap decreased both primary tumor volume in a TNBC xenograft model and almost completely abolished lung metastasis [118]. The effects of CRIPTO blockade seen in these studies appear to involved decreased CSC populations and reduced EMT [101,118,173], and notably involve signaling inhibition beyond CRIPTO/NODAL signaling as sulforaphane and Alk^L75A^-Fc can decrease tumor burden despite no apparent reduction in downstream CRIPTO/NODAL signaling [118,202].

A critical node in the CRIPTO signaling cascade in this regard may be GRP78. There is a substantial body of work investigating the effects of GRP78 inhibition on tumor growth. Although GRP78 is ubiquitously expressed, only a small fraction of GRP78 localizes to the cell surface when cells are challenged by ER stress. Surface expression of GRP78 is highly enriched in tumors but not in normal tissue [203]. A CRIPTO^high^ HCC patient-derived xenograft model was associated with resistance to the multi-protein kinase inhibitor sorafenib, and this effect could be modulated using a GRP78 blocking antibody. This suggests that a subgroup of CRIPTO-expressing HCC patients may benefit from a combinatorial treatment scheme and that sorafenib resistance may be circumventable by inhibition of GRP78 [96]. GRP78 has also been successfully used as a targeting agent for directed delivery of immunoconjugated chemotherapies in a murine xenograft model of HCC [204]. Direct targeting of surface GRP78 with a monoclonal antibody decreased primary tumor volume in pre-clinical models of colon, and lung adenocarcinomas [205]. In this study, there appeared to be an additive effect when GRP78 monoclonal antibodies were used in a combination with standard of care therapy in a colorectal xenograft model [205]. GRP78 therapeutics may be most effective in a combination setting in multi-drug resistant (MDR) tumors. Indeed, small molecule inhibition of GRP78 with a 4H-chromene derivative, CXL146, sensitized MDR malignant hematopoietic cells to early treatments [206]. Whether the effects seen with GRP78 inhibition are due exclusively to interactions with CRIPTO remains an important unanswered question as GRP78 has multiple putative client proteins [120].

## 7. Conclusions and Next Steps

### 7.1. Bridging the Gaps

In this review, we have highlighted recent advances and pressing questions in our understanding of CRIPTO’s biological roles and its mechanisms of action. Although much has been learned about CRIPTO in recent years, it has continued to live up to its name, which was assigned over 30 years ago when CRIPTO was discovered as an oncogene with unknown function. For instance, while directed analysis has revealed CRIPTO to be a stem cell regulatory factor that is frequently re-emergent in diverse cancers and concordantly shown it to be a clinically relevant biomarker of disease progression, unsupervised studies rarely identify CRIPTO and it remains difficult to detect in many settings where its ablation and blockade show significant effects (authors’ collective observations). While this likely reflects CRIPTO’s expression in a restricted subset of cells where it seems to have proportionally outsized roles in normal and disease physiology, it may nevertheless be critical to develop and employ improved, sensitive reagents and single cell techniques to definitively detect and distinguish CRIPTO and related factors in the experimental and clinical settings where CRIPTO acts. In this regard, the recent development of monoclonal CRIPTO-1 directed antibodies is a move in the right direction [207,208]. We also acknowledge that many studies to date, including several of our own, have relied on CRIPTO overexpression that may not reflect relevant physiological roles, and it could well be argued that CRISPR-based strategies have been underutilized. Indeed, complex regulation of multiple interacting signaling pathways (e.g., SMAD and AKT) likely rely on finely tuned CRIPTO levels and a better understanding of CRIPTO abundance and functional control at the transcriptional, translational, and especially post-translation levels (e.g., distinct glycosylation patterns in distinct systems) is needed. An in-depth study of local ligand concentrations in various tumor microenvironments as they impact CRIPTO-regulated signaling would likely also be illuminating. Thus, it remains unclear which signals ‘activate’ CRIPTO. Direct signaling readouts are also lacking. Live cell reporters usually comprise luciferase-based reporter constructs for SMAD and AKT activation (A3 and FOXO reporters, respectively), but these are rather indirect and both pathways are fraught with multiple activating and inhibitory upstream signaling inputs that are likely CRIPTO-independent. Furthermore, although its signaling through SMAD2/3 is well established, SMAD signaling may be dispensable for critical aspects of CRIPTO function and clinically effective CRIPTO-blockade. In addition, soluble versus cell attached CRIPTO pools may have different value as therapeutic targets. It will also be critical to identify proximal signaling mediators that enable the development of specific live cell signaling readouts. Such knowledge would also enable diversification of strategies for CRIPTO directed therapies.

Finally, the existing literature suggests that many of CRIPTO’s effects on cell phenotype are much more pronounced in vivo than they are in vitro. Yet, to date, the source and settings that specifically call upon CRIPTO signaling remain to be precisely elucidated. A key dependency on specific contexts, e.g., those involving metabolic and protein stress and/or micro-environmental factors such as fibrosis and inflammation as well as specific types of effector cells may help to explain CRIPTO’s mercurial nature and discrepancies between in vitro and in vivo studies. New models and approaches are needed that can resolve cellular heterogeneity and spatially delimited CRIPTO effects in vivo or reconstitute them in vitro. In this regard, primary heterotypic organoid cultures, stromally modified patient derived in vitro models and ‘organ on a chip’ approaches hold promise for deconvoluting CRIPTO function with relevant and interpretable yet sophisticated heterotypic signaling cells, key stromal effectors and tumor relevant modified environments that include cancer relevant stresses.

### 7.2. Forging Ahead

Despite the remaining challenges, recent advances have strengthened our interest and enthusiasm for eyeing CRIPTO as a viable therapeutic target in diverse neoplasms and inflammatory conditions. In some sense, CRIPTO satisfies the concept of an embryonic rest as proposed by Cohnhiem and Virchow some 140 years ago [209,210]. This theory held that cells from the embryo fail to differentiate, and instead remain in tissues as a tumor prone embryonic remnant or rests. However, rather than a persistent cell state, the oncofetal state that CRIPTO fosters may be facultative and highly context dependent, capable of being ‘rekindled’ by contextual ‘irritants’ as posited by Mueller even earlier (see Spike, 2016 [1]). Understanding and controlling CRIPTO function could thus provide a very satisfying end to a long-standing mystery.

Intrigue aside, controlling CRIPTO (and for that matter other pathways that similarly represent facultative, ‘stemness’-promoting, adaptive stress responses) could yield important clinical avenues for promoting effective allostasis and suppressing fibrosis, inflammation, dysplasia, neoplastic transformation and cancer progression. It is therefore with enthusiasm that we urge the field to press on and forge the tools needed to flush this unique therapeutic target into the open.

## Figures and Tables

**Table 1:** CRIPTO detection in normal and cancerous tissue.

Tissue Type	Expression in Normal Tissue	Expression in Cancer	Method of Detection
Blood	Detected in long-term HSCs [39]	-	RT-qPCR
Liver	Undetectable in healthy livers [35,40] Elevated expression in cirrhotic livers [35]	Overexpressed correlates with poor prognosis in HCC [41]	Northern blot, RT-qPCR
Mammary	Detectable in the surrounding fat pad of developing rudiments [42] Peak epithelial expression during alveogenesis [43,44]	Overexpressed in ~80% of invading breast cancers [45]	Immunohistochemistry RT-qPCR, Western
Muscle	Expressed in developing cardiac ventricles and outflow tracts [46] Upregulated in cardiac tissue post MI [35] Promotes skeletal muscle regeneration through myogenic differentiation of satellite cells and macrophage plasticity [37,47]	-	RNA in situ, Immunohistochemistry, FACS
CNS	Expressed throughout the CNS in adult non-human primates and upregulated during infection with simian human immunodeficiency virus [48].	Serum CRIPTO correlates with poor GBM prognosis. Localizes to perivascular tumor cells and endothelial cells in GMB [49]	ELISA, immunohistochemistry, RT-PCR
Pancreas	Moderate and low expression in normal ductal and acinar cells respectively. Increased expression in both ductal and acinar cells in chronic pancreatitis [50].	CRIPTO overexpression correlates with tumor stage [50]	Immunohistochemistry, RT-qPCR
Prostate	Low/undetectable level in healthy and BPH patient samples [51,52]	Metastatic lesions are characterized by CRIPTO overexpression [53] Overexpression in primary tumors predicts patient prognosis [51]	RT-qPCR, immunohistochemistry
Skin	Undetectable in healthy murine skin, upregulated in TPA treated murine skin and benign murine papillomas [54]	Overexpressed in a subset of cutaneous melanomas. Inversely correlates with saracatinib efficacy [55].	RT-qPCR
Renal	Low levels in adjacent non-tumor tissue relative to adjacent neoplastic lesions [56]	CRIPTO expression correlates with tumor aggressiveness and poor survival [56]	RT-qPCR
Colon	Undetectable in normal adult mucosa [57,58]	Detectable and independently correlates with poor prognosis [59]	Immunohistochemistry
Oral Squamous	Present sporadically at low levels in healthy tissue and widely expressed in dysplastic epithelia [60]	Overexpressed relative to homeostatic tissue [60]	Immunohistochemistry
Cervix	Low, variable expression across multiple healthy patient samples [61]	-	Immunohistochemistry
Gastric	Detectable in a subset of healthy patients [62,63]	Overexpressed relative to homeostatic tissue [62]	Immunohistochemistry
Bladder	-	Overexpressed relative to homeostatic tissue and correlated with tumor size and grade [64]	Immunohistochemistry
Lung	-	Overexpression predicts outcome in NSCLC [65]	Immunohistochemistry
Esophageal	Undetectable in normal adult epithelia [66]	Overexpressed in esophageal squamous cell carcinoma. Positively correlates with tumor stage [67].	Immunohistochemistry

## Data Availability

Not applicable.
